# Investigating the Mechanisms of Graded Sensorimotor Precision Training in Adults With Chronic Nonspecific Low Back Pain: Protocol for a Causal Mediation Analysis of the RESOLVE Trial

**DOI:** 10.2196/26053

**Published:** 2021-07-02

**Authors:** Aidan G Cashin, Hopin Lee, Matthew K Bagg, Benedict M Wand, Edel O'Hagan, Rodrigo R N Rizzo, Tasha R Stanton, G Lorimer Moseley, James H McAuley

**Affiliations:** 1 Prince of Wales Clinical School University of New South Wales Randwick Australia; 2 Centre for Pain IMPACT Neuroscience Research Australia Sydney Australia; 3 Oxford Clinical Trials Research Unit and Centre for Statistics in Medicine, Nuffield Department of Orthopaedics Rheumatology and Musculoskeletal Sciences University of Oxford Oxford United Kingdom; 4 School of Medicine and Public Health University of Newcastle Newcastle Australia; 5 School of Physiotherapy The University of Notre Dame Fremantle Australia; 6 School of Health Sciences University of New South Wales Randwick Australia; 7 IIMPACT in Health University of South Australia Adelaide Australia

**Keywords:** chronic low back pain, mediation analysis, mechanism evaluation, protocol

## Abstract

**Background:**

Chronic low back pain (CLBP) is a global health problem associated with an increasing burden on individuals, health care systems, and society. Common treatments for people with CLBP produce, on average, small short-term improvements in pain and function compared with minimal care. The RESOLVE trial randomly allocated 276 people with CLBP to a new complex treatment strategy, pain education integrated with graded sensorimotor precision training (RESOLVE), or a sham control. The RESOLVE treatment was developed within a theoretical framework to target possible treatment mechanisms associated with CLBP development and persistence.

**Objective:**

This protocol describes the planned evaluation of these proposed treatment mechanisms. Improved understanding of the mechanisms underpinning the RESOLVE treatment may guide its refinement and implementation.

**Methods:**

We will use causal mediation analysis to evaluate the proposed treatment mechanisms, including pain self-efficacy, back beliefs, pain catastrophizing, kinesiophobia, back perception, tactile acuity, and movement coordination. The primary outcomes are pain intensity and function at 18 weeks following allocation. Data were collected blind to allocation and hypotheses at baseline (mediators, outcomes, confounders), end of treatment (mediators), and at 18 weeks following allocation (outcomes). We will test the robustness of our findings by conducting planned sensitivity analyses.

**Results:**

Ethical approval was granted by the University of New South Wales Human Research Ethics Committee (HC15357). A total of 276 participants have been recruited from primary care practices and the community in Sydney, Australia.

**Conclusions:**

The RESOLVE treatment constitutes a new paradigm for CLBP management with potentially wide-reaching implications. This mechanistic evaluation will provide evidence for the hypothesized treatment mechanisms and help explain why the treatment strategy did or did not have an effect on patient-reported outcomes. These results will help guide the treatment refinement and implementation.

**Trial Registration:**

Australian and New Zealand Clinical Trials Registry ACTRN12615000610538; https://www.anzctr.org.au/Trial/Registration/TrialReview.aspx?id=368619&isReview=true

**International Registered Report Identifier (IRRID):**

DERR1-10.2196/26053

## Introduction

Low back pain (LBP) is a global health problem [1,2]. The associated personal and societal burden continues to increase, despite the increasing amount of health care resources devoted to LBP treatment [3,4]. Although many recover from a new episode of LBP, recurrence is common, and for a small proportion pain becomes persistent and significantly disabling [5-7]. Individuals who develop chronic low back pain (CLBP) have a reduced chance of recovery and experience substantial functional limitations and poor quality of life [8,9].

People with LBP perceive recovery as a complex interaction of decreased pain, improved function, and reduced symptom interference with daily life [10]. Common treatments provide mostly small short-term improvements in pain and function, when compared with minimal care [11,12]. Demand for improved treatment effects is pressing. There is a limited understanding of why common treatments are ineffective and a lack of high-quality evidence on promising new treatment targets [13]. Better evidence regarding the mechanisms of treatments can help address these problems and has been identified by pain researchers as one of the highest research priorities [14].

A clearer understanding of the biopsychosocial influences on pain has promoted the development of new explanatory models for CLBP [15] and new treatment strategies [16-19]. Accumulating evidence demonstrates structural, functional, and biochemical differences in the central nervous system between people with CLBP and people without pain [20], many of which appear related to the CLBP experience [21-23]. The RESOLVE trial is a randomized controlled trial (RCT) evaluating a new complex treatment strategy (pain education integrated with graded sensorimotor precision training) partly informed by evidence of central nervous system dysfunction against a sham control.

People with CLBP want improvements in pain and function [10], and the effects of the RESOLVE treatment on these key outcomes will be evaluated [24]. Yet these effect estimates will not elucidate the mechanisms through which the effects occurred. We present an a priori protocol for a secondary analysis of the RESOLVE trial to estimate the effects of the RESOLVE treatment on 7 proposed treatment mechanisms, and to estimate whether these mechanisms cause change in pain and function. The aim of this study is to evaluate these effects through a causal mediation analysis to guide treatment optimization and implementation.

## Methods

### Design

The study involves a causal mediation analysis of a 2-group participant and assessor-blinded RCT [24,25]. The RESOLVE trial was prospectively registered (ACTRN12615000610538) and approved by the University of New South Wales Human Research Ethics Committee (HC15357).

### Participants

Participants were recruited from primary care practices and the community in Sydney, Australia. The eligibility criteria are comprehensively described in the trial protocol [25]. Briefly, the RESOLVE trial included people reporting nonspecific LBP [26] (intensity rated at least 3/10), with or without leg pain, that had persisted for at least 12 consecutive weeks. Participants were aged between 18 and 70, fluent in English, able to access the internet, and had a trusted person to assist with the home portion of the intervention. The RESOLVE trial excluded people with LBP due to serious pathology, and people with contraindications to physical activity, transcranial direct current stimulation, cranial electrical simulation, low-intensity laser therapy, or short-wave diathermy. Finally, the RESOLVE trial excluded people who were pregnant or had given birth in the previous 6 months, had undergone spinal surgery in the previous 12 months, were scheduled for major surgery in the next 12 months, or had an uncontrolled mental health condition that would impede participation.

### Randomization

Eligible participants were randomly allocated in a 1:1 ratio to the RESOLVE treatment or the sham-control treatment. The allocation schedule was generated a priori by a scientist independent to the trial using blocked randomization. The allocations were printed and placed in 276 sealed, opaque, sequentially numbered envelopes. Participants and assessors were blind to both group allocation and study hypotheses throughout the trial and follow-up period.

### Interventions

The treatments are comprehensively described in the trial protocol [25] and briefly here. Each treatment group received twelve 30-60-minute one-on-one treatment sessions with a clinician, scheduled approximately weekly over 12-18 weeks. The treatment sessions were supplemented with a home treatment component entailing 30 minutes of training 5 times per week. Concomitant interventions were allowed and recorded on a weekly treatment diary.

### RESOLVE Treatment Group

The RESOLVE treatment comprised 4 treatment components delivered with a standard progression protocol. The components were pain education, graded sensory training, movement simulation training, and graded precision-focused feedback-enriched functional movement training. The intent was to help people in pain *understand* that it is safe and helpful to move, *feel* safe to move, and *experience* safety with movement as they progress toward reengagement with meaningful functional goals.

Pain education was delivered throughout the treatment period to improve the participants’ understanding of pain and their CLBP problem, address maladaptive beliefs, improve engagement with treatment, and emphasize the value of movement and physical activity. The pain education was based on the *Explain Pain* model [27,28], delivered according to a standard curriculum, and individualized to the patients lived experience and CLBP narrative. The educational material was delivered by the study clinicians and included the use of graphical media, video, metaphor, and narrative [27,29,30].

Sensory precision training comprised tactile localization training, discrimination of sharp/blunt sensations, and graphesthesia training. The movement simulation component was grounded in graded motor imagery, developed for pathological limb pain [31], and included left–right recognition training using the Recognise software [32] and implicit and explicit motor imagery training using a series of custom-designed videos on low back movements.

Graded precision-focused feedback-enriched functional movement training included individualized movement training related to the patients’ goals. Training progressed from part practice to whole task practice within a visual and proprioceptive feedback-enriched environment [33].

### Sham Treatment Group

The sham treatment was composed of 3 treatment components to match for time and clinician interaction, individualization, and relevance [34]. These include passive discussion of the participant’s back pain experience, detuned low-intensity laser therapy, and detuned short-wave diathermy. Participants also received a home training program of sham cranial electrical stimulation to control for the home training requirements of the RESOLVE treatment group.

### Mediators, Outcomes, and Confounder

#### Overview

Patient characteristics, outcome measures, mediators, and potential confounders were assessed at baseline. Mediators were assessed again following the twelfth treatment session, approximately 12-18 weeks following allocation. Outcome measures were assessed again 18 weeks following allocation. Participants and outcome assessors were blind to group allocation and study hypotheses.

#### Outcomes

We will consider 2 primary outcome measures for this mediation study:

1. Average pain intensity over the past week, assessed using an 11-point Numeric Rating Scale (NRS; 0=no pain, 10=pain as bad as it could be) [35], considered a valid, reliable, and responsive measure of pain intensity [36].
2. Function, assessed using the 24-item Roland–Morris Disability Questionnaire [37], considered a valid and reliable measure of low back–related disability [38,39].

#### Mediators

**Figure 1:**
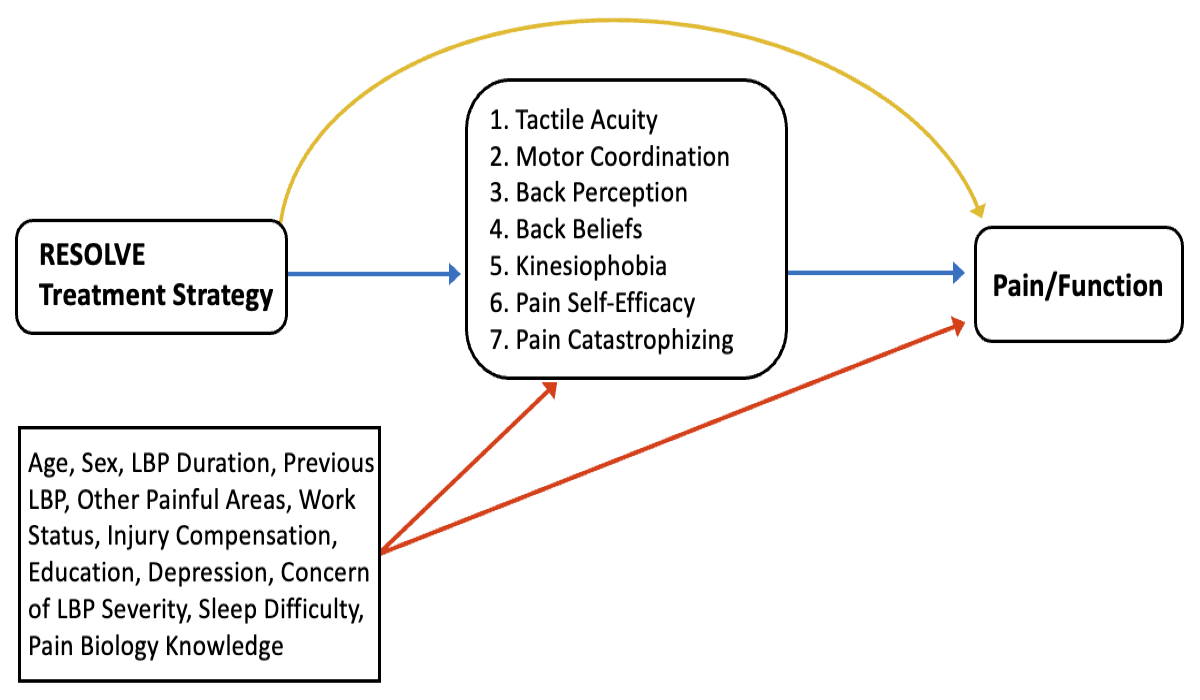
Simplified causal pathways for the effect of the RESOLVE treatment on the outcomes, pain intensity and function, via the hypothesized mediators. The mediators are measured at the end of treatment. Outcomes are measured at 18 weeks after randomization. The potential confounders are measured at baseline. The diagram assumes independence of mediators. The treatment–mediator relationship is represented by the blue line from the RESOLVE treatment to the mediators. The mediator–outcome relationship is represented by the blue line from the mediators to the outcomes. The potential confounders of the mediator–outcome relationship are represented by the red lines. The direct effect of treatment on the outcome is represented by the yellow line.

We will investigate 7 hypothesized mediators. A simplified model of the hypothesized causal relationships between the effects of the RESOLVE treatment on the outcomes through the mediator(s) is presented in Figure 1.

The 7 mediators to be modeled are as follows:

1. Tactile acuity: measured using a digital caliper to establish 2-point discrimination thresholds over the lumbar region of most discomfort [40], considered a reliable measure of tactile acuity [41].
2. Lumbopelvic motor coordination: measured on a clinical scale assessing the ability to dissociate lumbopelvic movement from that of the thoracolumbar junction, considered a reliable measure of lumbopelvic control when assessed by an experienced clinician/assessor [42].
3. Back-specific body perception: assessed using the Fremantle Back Awareness Questionnaire (FreBAQ) [43]. The FreBAQ has 9 items, each scored on a 5-point scale (0=Never, 4=Always). The total score ranges from 0 to 36, with higher scores indicating higher body perceptual disturbance. The FreBAQ is considered a psychometrically sound method for assessing disruption of body image in people with CLBP [43].
4. Back beliefs: assessed on the Back Beliefs Questionnaire (BBQ) [44]. The BBQ has 14 items, including 5 distractors, each scored on a 5-point Likert scale (1=completely agree, 5=completely disagree). The total score ranges from 9 to 45, with lower scores indicating more pessimistic beliefs about the consequences of LBP. The BBQ is a valid and reliable measure to quantify beliefs about the consequences of LBP [45].
5. Fear of movement-related pain (kinesiophobia): assessed on the Tampa Scale for Kinesiophobia (TSK) [46]. The TSK has 17 items, each scored on a 4-item scale (1=strongly disagree, 4=strongly agree). The total score ranges from 17 to 68, with higher scores indicating greater levels of fear of movement-related pain. The TSK is a reliable and valid measurement tool that provides information on activity avoidance and pathological somatic focus [47].
6. Pain-related self-efficacy: assessed on the Pain Self-Efficacy Questionnaire (PSEQ) [48]. The PSEQ has 10 items, scored on a 7-point Likert scale (0=not confident at all, 6=completely confident). The total score ranges from 0 to 60, with higher scores indicating greater confidence in the ability to undertake activities despite pain. The PSEQ has adequate psychometric properties [49,50].
7. Pain catastrophizing: assessed on the Pain Catastrophizing Scale (PCS) [51]. The PCS has 13 items, scored on a 5-point scale (0=not at all, 4=all the time). The total score ranges from 0 to 52, with higher scores indicating an exaggerated perception of pain-related problems. The PCS is a reliable measure to assess catastrophic thoughts about pain [51,52].

#### Confounders

We assumed no confounding of the treatment–mediator and treatment–outcome relationships due to random allocation of treatment. We identified potential confounders of the mediator–outcome relationship using the disjunctive cause criterion [53,54]. This involved selecting measured pretreatment covariates that are hypothesized to be a cause of the mediator, outcome, or both. The minimum sufficient adjustment set includes age, biological sex, duration of LBP episode, number of previous LBP episodes, number of other painful areas, work status, injury compensation, education level, depression, concern of LBP severity, sleep difficulty, and pain biology knowledge (refer to Multimedia Appendix 1 for details on potential confounders). We will also include pretreatment measures of the mediators and outcomes in the models [55].

### Causal Mediation Analysis: Rationale

We will test the mechanisms of the RESOLVE treatment strategy for adults with CLBP by estimating the extent to which the 7 hypothesized mediators explain the effect of the treatment on the participants’ pain and disability scores. Using causal mediation analysis, we will partition the total effect (TE) of the treatment into an indirect effect which operates through the mechanism(s) of interest, and a direct effect which operates through all other possible mechanisms [56,57].

The RESOLVE treatment strategy was designed around the Maladaptive Perceptions Model [58], an explanatory framework for the development and persistence of LBP that is grounded in a broad scope of literature concerning behavioral (eg, movement avoidance), neurobiological (eg, altered cortical representations), and cognitive (unhelpful and inaccurate beliefs about the biology of pain and the structural integrity of the back) characteristics of CLBP [20]. The RESOLVE treatment integrates contemporary understandings of pain with known features of best practice care to address the biopsychosocial contributors to the CLBP experience, including maladaptive conceptualizations of the pain problem [59-61], altered sensory function [62,63], altered motor function [64-68], and altered self-perception of the back [43].

While the causal mechanisms that underpin improvement or recovery from CLBP are not well established [13], the Maladaptive Perceptions Model [58] proposes several intermediary variables through which effects might occur. These are cognitions about the back, pain, and movement [69]; back-specific body representations [43]; fidelity and weighting of sensory information from the back [70,71]; and spinal control, movement coordination, and functional tolerance for meaningful activities [33,72]. Components of the RESOLVE treatment were designed to target these factors alongside pain and function.

We have chosen the proposed mediators, pain self-efficacy, back beliefs, pain catastrophizing, kinesiophobia, back perception, tactile acuity, and movement coordination, based on theoretical predictions from the Maladaptive Perceptions Model [58] and the results of pilot studies [33,69-71,73,74].

### Effects of Interest

We will estimate the effect and corresponding uncertainty for the treatment–mediator relationship. This is the average unstandardized effect of the RESOLVE treatment on each independent mediator, compared with sham control. We will also estimate the effect and corresponding uncertainty for the mediator–outcome relationship. This is the average unstandardized effect of the mediator on the outcome (Figure 1).

If inference is considered feasible given the causal assumptions, we will also estimate natural (in)direct effects of the RESOLVE treatment on the outcomes considering (1) the mediators independently, while assuming independence of the mediators, and (2) the mediators simultaneously as a joint mediator.

### Causal Model

The identification of natural (in)direct effects relies on several strong and untestable causal assumptions including (1) no treatment–outcome confounding, (2) no mediator–outcome confounding, (3) no treatment–mediator confounding, and (4) no mediator–outcome confounder that is itself affected by the treatment [75]. Adjustment for a sufficient set of observed confounders and correct specification of the statistical models may provide sufficient conditions to identify mediation effects and causal interpretation [76]. Assumption (4) may not hold because there are possible causal relationships between mediators. We will also assess the mediators simultaneously as a joint mediator which relies on weaker assumptions for identification [77,78]. The causal model is presented in Figure 1.

### Statistical Analysis

Analyses will be performed in R (version 3.6.1; R Foundation for Statistical Computing) [79]. We will use the “mediation” package [80] to estimate independent mediated effects and the “medflex” package [78] to estimate joint mediated effects.

We will estimate effects for the treatment–mediator and mediator–outcome relationship with 2 regression models: the mediator model and the outcome model. We will specify the mediator model as a linear regression of the mediator (dependent variable) on treatment allocation and baseline values of the mediator. The outcome model will be specified as a linear regression of the outcome at 18 weeks (dependent variable) on the mediator at baseline and follow up, treatment allocation, and possible confounders of the mediator-outcome relationship, and a treatment allocation x mediator interaction term. We will also include an interaction term (treatment allocation × mediator) in the outcome models to increase model flexibility [57].

### Independent Mediated Effects

A model-based inference approach developed by Imai et al [81] will be used to estimate independent mediated effects for each mediator. We will use the “mediate” function [80] to compute the average treatment effect, the average causal mediation effect (ACME), and the average direct effect (ADE). We will use 1000 bootstrapped simulations to estimate 95% CIs. We will interpret conditional estimates of the ACME and ADE separately if there is evidence for a significant (*P*<.05) intervention–mediator interaction. If there is no evidence for an interaction, we will interpret the average of the conditional effects for the ACME and ADE.

### Joint Mediated Effects

An imputation-based approach using a class of natural effect models introduced by Lange et al [82] and Vansteelandt et al [83] will be used to estimate the joint mediated effect of all mediators simultaneously. We will use the “neModel” function [78] to compute the natural indirect effect (NIE), natural direct effect (NDE), and the TE. We will use 1000 bootstrapped simulations to estimate 95% CIs.

### Missing Data

We will assess the proportion and patterns of missing mediator and outcome data. We will conduct all analyses on complete cases if the proportion of missing data is less than 15% for all variables in a given model. If missing data exceed 15%, we will impute missing data through multiple imputation by chain equations using the “mice” package [84] in R.

### Sensitivity Analyses

We will conduct sensitivity analyses to determine the robustness of the estimated ACME to bias introduced by unmeasured pretreatment confounding in the independent mediated effect models [85]. We will use the “medsens” function [80] to estimate the magnitude of residual confounding that would cause the point estimate of the ACME to reach 0. The level of residual confounding is represented by the correlation between the residuals (error terms) in the mediator and outcome models, denoted *ρ*. By estimating the ACME, including point estimates and 95% CIs, at all possible levels of *ρ* (between the extremes of –1 and +1), we can determine how strong the effects of residual confounding would need to be to reduce the ACME to 0 (ie, no mediating effect).

We will conduct a sensitivity analysis to determine the robustness of the estimated NIE and NDE to possible unmeasured confounding in the joint mediated effect model [85]. The mediational E-value [86] will be used to assess the minimum strength of the relationship between an unmeasured confounder and the mediator, conditional on measured confounders, that would reduce the NIE and NDE to 0. A comparatively large E-value in relation to known confounders implies that considerable unmeasured confounding would be required to reduce the NIE and NDE to 0. A comparatively small E-value implies that little unmeasured confounding would be required to reduce the NIE and NDE to 0. We will use the “EValue” package [87] in R to estimate the mediational E-value.

If appropriate, we will conduct a sensitivity analysis to assess possible violations to the temporal ordering of the mediator–outcome relationship, excluding participants for whom the mediators and outcomes were assessed concurrently at 18 weeks.

### Secondary Analyses

If there is evidence of a mediator–outcome effect, we will investigate the magnitude of change required in the mediator(s) to produce a minimally clinically important difference (MCID) in pain intensity (of 1 point out of 10) [88] and disability (of 2 points out of 24) [89].

### Ethics Approval and Consent to Participate

The University of New South Wales Human Research Ethics Committee granted ethical approval (HC15357), and all participants provided written informed consent to participate.

## Results

A total of 276 participants have been randomized into the RESOLVE trial. Follow-up data collection is underway with authors blind to study data.

## Discussion

We present an analysis plan for a mechanism evaluation of a new complex treatment strategy combining pain education and graded sensorimotor precision training (RESOLVE), when compared with a sham treatment in people with CLBP. The RESOLVE treatment constitutes a new paradigm for CLBP management with potentially wide-reaching implications. This mechanism evaluation will provide evidence for the hypothesized treatment mechanisms. If the treatment is effective, this investigation will help explain how the treatment worked, and if the treatment is ineffective, it will help explain why the treatment did not work. These results will help adapt and refine the treatment and guide future implementation strategies.

## Appendix

Multimedia Appendix 1 Potential confounders.

## Abbreviations

ACME: average causal mediation effect
ADE: average direct effect
ATE: average total effect
BBQ: Back Beliefs Questionnaire
CLBP: chronic low back pain
FreBAQ: Fremantle Back Awareness Questionnaire
LBP: low back pain
MCID: minimally clinically important difference
NDE: natural direct effect
NIE: natural indirect effect
NRS: Numeric Rating Scale
PCS: Pain Catastrophizing Scale
PSEQ: Pain Self-Efficacy Questionnaire
RCT: randomized controlled trial
TE: total effect
TSK: Tampa Scale for Kinesiophobia

## References

[1] Hartvigsen J, Hancock MJ, Kongsted A, Louw Q, Ferreira ML, Genevay S, Hoy D, Karppinen J, Pransky G, Sieper J, Smeets RJ, Underwood M, Lancet Low Back Pain Series Working Group (2018). What low back pain is and why we need to pay attention. Lancet.

[2] GBD 2017 Disease Injury Incidence Prevalence Collaborators (2018). Global, regional, and national incidence, prevalence, and years lived with disability for 354 diseases and injuries for 195 countries and territories, 1990-2017: a systematic analysis for the Global Burden of Disease Study 2017. Lancet.

[3] Freburger JK, Holmes GM, Agans RP, Jackman AM, Darter JD, Wallace AS, Castel LD, Kalsbeek WD, Carey TS (2009). The rising prevalence of chronic low back pain. Arch Intern Med.

[4] Hoy D, March L, Brooks P, Woolf A, Blyth F, Vos T, Buchbinder R (2010). Measuring the global burden of low back pain. Best Practice & Research Clinical Rheumatology.

[5] da Silva T, Mills K, Brown BT, Herbert RD, Maher CG, Hancock MJ (2017). Risk of recurrence of low back pain: a systematic review. J Orthop Sports Phys Ther.

[6] Itz C, Geurts J, van Kleef M, Nelemans P (2012). Clinical course of non-specific low back pain: a systematic review of prospective cohort studies set in primary care. EJP.

[7] da C Menezes Costa L, Maher CG, Hancock MJ, McAuley JH, Herbert RD, Costa LOP (2012). The prognosis of acute and persistent low-back pain: a meta-analysis. CMAJ.

[8] Bunzli S, Watkins R, Smith A, Schütze R, O'Sullivan P (2013). Lives on hold: a qualitative synthesis exploring the experience of chronic low-back pain. Clin J Pain.

[9] Schofield DJ, Callander EJ, Shrestha RN, Percival R, Kelly SJ, Passey ME (2012). Labor force participation and the influence of having back problems on income poverty in Australia. Spine (Phila Pa 1976).

[10] Hush JM, Refshauge K, Sullivan G, De Souza L, Maher CG, McAuley JH (2009). Recovery: what does this mean to patients with low back pain?. Arthritis Rheum.

[11] Machado LAC, Kamper SJ, Herbert RD, Maher CG, McAuley JH (2009). Analgesic effects of treatments for non-specific low back pain: a meta-analysis of placebo-controlled randomized trials. Rheumatology (Oxford).

[12] Foster NE, Anema JR, Cherkin D, Chou R, Cohen SP, Gross DP, Ferreira PH, Fritz JM, Koes BW, Peul W, Turner JA, Maher CG, Lancet Low Back Pain Series Working Group (2018). Prevention and treatment of low back pain: evidence, challenges, and promising directions. Lancet.

[13] Lee H, Mansell G, McAuley J, Kamper S, Hübscher M, Moseley G, Wolfenden L, Hodder R, Williams C (2016). Causal mechanisms in the clinical course and treatment of back pain. Best Practice & Research Clinical Rheumatology.

[14] Costa LDCM, Koes BW, Pransky G, Borkan J, Maher CG, Smeets RJEM (2013). Primary care research priorities in low back pain: an update. Spine (Phila Pa 1976).

[15] Moseley GL, Butler DS (2017). Explain Pain Supercharged.

[16] Wand BM, O'Connell NE, Di Pietro F, Bulsara M (2011). Managing chronic nonspecific low back pain with a sensorimotor retraining approach: exploratory multiple-baseline study of 3 participants. Phys Ther.

[17] Wälti P, Kool J, Luomajoki H (2015). Short-term effect on pain and function of neurophysiological education and sensorimotor retraining compared to usual physiotherapy in patients with chronic or recurrent non-specific low back pain, a pilot randomized controlled trial. BMC Musculoskelet Disord.

[18] Malfliet A, Kregel J, Coppieters I, De Pauw R, Meeus M, Roussel N, Cagnie B, Danneels L, Nijs J (2018). Effect of pain neuroscience education combined with cognition-targeted motor control training on chronic spinal pain. JAMA Neurol.

[19] Schabrun SM, Jones E, Elgueta Cancino EL, Hodges PW (2014). Targeting chronic recurrent low back pain from the top-down and the bottom-up: a combined transcranial direct current stimulation and peripheral electrical stimulation intervention. Brain Stimulation.

[20] Wand BM, Parkitny L, O'Connell NE, Luomajoki H, McAuley JH, Thacker M, Moseley GL (2011). Cortical changes in chronic low back pain: current state of the art and implications for clinical practice. Man Ther.

[21] Malfliet A, Coppieters I, Van Wilgen P, Kregel J, De Pauw R, Dolphens M, Ickmans K (2017). Brain changes associated with cognitive and emotional factors in chronic pain: A systematic review. Eur J Pain.

[22] Coppieters I, Meeus M, Kregel J, Caeyenberghs K, De Pauw Robby, Goubert D, Cagnie B (2016). Relations between brain alterations and clinical pain measures in chronic musculoskeletal pain: a systematic review. J Pain.

[23] Goossens N, Rummens S, Janssens L, Caeyenberghs K, Brumagne S (2018). Association between sensorimotor impairments and functional brain changes in patients with low back pain. Am J Phys Med Rehabil.

[24] Bagg MK, Lo S, Cashin AG, Herbert RD, O'Connell NE, Lee H, Hübscher M, Wand BM, O'Hagan E, Rizzo RR, Moseley GL, Stanton TR, Maher CG, Goodall S, Saing S, McAuley JH (2021). The RESOLVE Trial for people with chronic low back pain: statistical analysis plan. Braz J Phys Ther.

[25] Bagg MK, Hübscher M, Rabey M, Wand BM, O'Hagan E, Moseley GL, Stanton TR, Maher CG, Goodall S, Saing S, O'Connell NE, Luomajoki H, McAuley JH (2017). The RESOLVE Trial for people with chronic low back pain: protocol for a randomised clinical trial. J Physiother.

[26] Maher C, Underwood M, Buchbinder R (2017). Non-specific low back pain. Lancet.

[27] Butler DS, Moseley GL (2013). Explain Pain (2nd Edition).

[28] Moseley GL, Butler DS (2015). Fifteen years of explaining pain: the past, present, and future. J Pain.

[29] Moseley GL, Butler DS (2015). The Explain Pain Handbook: Protectometer.

[30] Moseley GL, Butler DS, Beames TB, Giles TJ (2012). The Graded Motor Imagery Handbook.

[31] Moseley GL (2004). Graded motor imagery is effective for long-standing complex regional pain syndrome: a randomised controlled trial. Pain.

[32] Recognise App. NOIgroup.

[33] Wand BM, Tulloch VM, George PJ, Smith AJ, Goucke R, O'Connell NE, Moseley GL (2012). Seeing it helps: movement-related back pain is reduced by visualization of the back during movement. Clin J Pain.

[34] Machado LAC, Kamper SJ, Herbert RD, Maher CG, McAuley JH (2008). Imperfect placebos are common in low back pain trials: a systematic review of the literature. Eur Spine J.

[35] Kamper SJ (2012). Pain intensity ratings. J Physiother.

[36] Dworkin RH, Turk DC, Farrar JT, Haythornthwaite JA, Jensen MP, Katz NP, Kerns RD, Stucki G, Allen RR, Bellamy N, Carr DB, Chandler J, Cowan P, Dionne R, Galer BS, Hertz S, Jadad AR, Kramer LD, Manning DC, Martin S, McCormick CG, McDermott MP, McGrath P, Quessy S, Rappaport BA, Robbins W, Robinson JP, Rothman M, Royal MA, Simon L, Stauffer JW, Stein W, Tollett J, Wernicke J, Witter J, IMMPACT (2005). Core outcome measures for chronic pain clinical trials: IMMPACT recommendations. Pain.

[37] Roland M, Morris R (1983). A study of the natural history of back pain. Part I: development of a reliable and sensitive measure of disability in low-back pain. Spine (Phila Pa 1976).

[38] Roland M, Fairbank J (2000). The Roland-Morris Disability Questionnaire and the Oswestry Disability Questionnaire. Spine (Phila Pa 1976).

[39] Stevens ML, Lin CC, Maher CG (2016). The Roland Morris Disability Questionnaire. J Physiother.

[40] Catley MJ, Tabor A, Wand BM, Moseley GL (2013). Assessing tactile acuity in rheumatology and musculoskeletal medicine--how reliable are two-point discrimination tests at the neck, hand, back and foot?. Rheumatology (Oxford).

[41] Cashin AG, McAuley JH (2017). Measuring two-point discrimination threshold with a caliper. J Physiother.

[42] Elgueta-Cancino E, Schabrun S, Danneels L, Hodges P (2014). A clinical test of lumbopelvic control: development and reliability of a clinical test of dissociation of lumbopelvic and thoracolumbar motion. Man Ther.

[43] Wand BM, Catley MJ, Rabey MI, O'Sullivan PB, O'Connell NE, Smith AJ (2016). Disrupted self-perception in people with chronic low back pain. Further evaluation of the Fremantle Back Awareness Questionnaire. J Pain.

[44] Symonds TL, Burton AK, Tillotson KM, Main CJ (1996). Do attitudes and beliefs influence work loss due to low back trouble?. Occup Med (Lond).

[45] Ferreira GE, Kamper SJ (2020). Clinimetrics: The Back Beliefs Questionnaire. J Physiother.

[46] Miller RP, Kori SH, Todd DD (1991). The Tampa Scale: a Measure of Kinisophobia. Clin J Pain.

[47] Weermeijer JD, Meulders A (2018). Clinimetrics: Tampa Scale for Kinesiophobia. J Physiother.

[48] Nicholas M (1989). Self-efficacy and chronic pain.

[49] Nicholas MK (2007). The pain self-efficacy questionnaire: Taking pain into account. Eur J Pain.

[50] Di Pietro Flavia, Catley M, McAuley JH, Parkitny L, Maher CG, Costa LD, Macedo LG, Williams CM, Moseley GL (2014). Rasch analysis supports the use of the Pain Self-Efficacy Questionnaire. Phys Ther.

[51] Sullivan MJL, Bishop SR, Pivik J (1995). The Pain Catastrophizing Scale: Development and validation. Psychol Assess.

[52] Osman A, Barrios FX, Gutierrez PM, Kopper BA, Merrifield T, Grittmann L (2000). The Pain Catastrophizing Scale: further psychometric evaluation with adult samples. J Behav Med.

[53] VanderWeele TJ, Shpitser I (2011). A new criterion for confounder selection. Biometrics.

[54] VanderWeele TJ (2019). Principles of confounder selection. Eur J Epidemiol.

[55] Landau S, Emsley R, Dunn G (2015). Beyond total treatment effects in RCTs: why we need to measure outcomes at baseline when investigating mediation. Trials.

[56] Lee H, Herbert RD, McAuley JH (2019). Mediation Analysis. JAMA.

[57] Vanderweele T (2015). Explanation in Causal Inference.

[58] Wand BM (2012). Chronic Lower Back Pain: A Maladaptive Perceptions Model.

[59] Crombez G, Vlaeyen JW, Heuts PH, Lysens R (1999). Pain-related fear is more disabling than pain itself: evidence on the role of pain-related fear in chronic back pain disability. Pain.

[60] Main CJ, Foster N, Buchbinder R (2010). How important are back pain beliefs and expectations for satisfactory recovery from back pain?. Best Pract Res Clin Rheumatol.

[61] Darlow B, Dean S, Perry M, Mathieson F, Baxter GD, Dowell A (2015). Easy to harm, hard to heal: patient views about the back. Spine.

[62] Hashmi J, Baliki M, Huang L, Baria A, Torbey S, Hermann K, Schnitzer TJ, Apkarian AV (2013). Shape shifting pain: chronification of back pain shifts brain representation from nociceptive to emotional circuits. Brain.

[63] Schneider C, Palomba D, Flor H (2004). Pavlovian conditioning of muscular responses in chronic pain patients: central and peripheral correlates. Pain.

[64] Flor H, Braun C, Elbert T, Birbaumer N (1997). Extensive reorganization of primary somatosensory cortex in chronic back pain patients. Neurosci Lett.

[65] Catley MJ, O'Connell NE, Berryman C, Ayhan FF, Moseley GL (2014). Is tactile acuity altered in people with chronic pain? a systematic review and meta-analysis. J Pain.

[66] Moseley LG (2008). I can't find it! Distorted body image and tactile dysfunction in patients with chronic back pain. Pain.

[67] Tsao H, Danneels LA, Hodges PW (2011). ISSLS prize winner: Smudging the motor brain in young adults with recurrent low back pain. Spine (Phila Pa 1976).

[68] Bray H, Moseley GL (2011). Disrupted working body schema of the trunk in people with back pain. Br J Sports Med.

[69] Moseley GL, Nicholas MK, Hodges PW (2004). A randomized controlled trial of intensive neurophysiology education in chronic low back pain. Clin J Pain.

[70] Wand BM, Abbaszadeh S, Smith AJ, Catley MJ, Moseley GL (2013). Acupuncture applied as a sensory discrimination training tool decreases movement-related pain in patients with chronic low back pain more than acupuncture alone: a randomised cross-over experiment. Br J Sports Med.

[71] Louw A, Farrell K, Wettach L, Uhl J, Majkowski K, Welding M (2015). Immediate effects of sensory discrimination for chronic low back pain: a case series. NZJP.

[72] Gardner T, Refshauge K, McAuley JH, Hübscher M, Goodall S, Smith L (2019). Combined education and patient-led goal setting intervention reduced chronic low back pain disability and intensity at 12 months: a randomised controlled trial. Br J Sports Med.

[73] Diers M, Löffler A, Zieglgänsberger W, Trojan J (2015). Watching your pain site reduces pain intensity in chronic back pain patients. Eur J Pain.

[74] Trapp W, Weinberger M, Erk S, Fuchs B, Mueller M, Gallhofer B, Hajak G, Kübler A, Lautenbacher S (2015). A brief intervention utilising visual feedback reduces pain and enhances tactile acuity in CLBP patients. BMR.

[75] VanderWeele TJ (2016). Mediation Analysis: A Practitioner's Guide. Annu Rev Public Health.

[76] Vanderweele T, Vansteelandt S (2009). Conceptual issues concerning mediation, interventions and composition. Stat Interface.

[77] VanderWeele T, Vansteelandt S (2014). Mediation analysis with multiple mediators. Epidemiol Methods.

[78] Steen J, Loeys T, Moerkerke B, Vansteelandt S (2017). Flexible mediation analysis with multiple mediators. Am J Epidemiol.

[79] (2013). R: A language and environment for statistical computing Internet.

[80] Tingley D, Yamamoto T, Hirose K, Keele L, Imai K (2014). Mediation: R package for causal mediation analysis. J Stat Soft.

[81] Imai K, Keele L, Tingley D (2010). A general approach to causal mediation analysis. Psychol Methods.

[82] Lange T, Vansteelandt S, Bekaert M (2012). A simple unified approach for estimating natural direct and indirect effects. Am J Epidemiol.

[83] Vansteelandt S, Bekaert M, Lange T (2012). Imputation strategies for the estimation of natural direct and indirect effects. Epidemiol Method.

[84] Buuren SV, Groothuis-Oudshoorn K (2011). mice: multivariate imputation by chained equations in R. J Stat Soft.

[85] Imai K, Keele L, Yamamoto T (2010). Identification, inference and sensitivity analysis for causal mediation effects. Statist Sci.

[86] Smith LH, VanderWeele TJ (2019). Mediational E-values: approximate sensitivity analysis for unmeasured mediator-outcome confounding. Epidemiology.

[87] Mathur MB, Ding P, Riddell CA, VanderWeele TJ (2018). Web site and R package for computing E-values. Epidemiology.

[88] Busse JW, Bartlett SJ, Dougados M, Johnston BC, Guyatt GH, Kirwan JR, Kwoh K, Maxwell LJ, Moore A, Singh JA, Stevens R, Strand V, Suarez-Almazor ME, Tugwell P, Wells GA (2015). Optimal strategies for reporting pain in clinical trials and systematic reviews: recommendations from an OMERACT 12 workshop. J Rheumatol.

[89] Bombardier C, Hayden J, Beaton DE (2001). Minimal clinically important difference. Low back pain: outcome measures. J Rheumatol.

