# Supplementary material for: Investigating the Mechanisms of Graded Sensorimotor Precision Training in Adults With Chronic Nonspecific Low Back Pain: Protocol for a Causal Mediation Analysis of the RESOLVE Trial
Source: JMIR Res Protoc. 2021 Jul 2;10(7):e26053. doi: 10.2196/26053 (PMC8285749; doi:10.2196/26053)
Supplement: Multimedia Appendix 1 [file resprot_v10i7e26053_app1.docx]

**SUPPLEMENTARY FILES**

**Supplementary Table 1.** Potential pre-treatment confounders of the mediator-outcome relationship

| **Confounder** | **Variable type** | **Description** |
| --- | --- | --- |
| 1. Age | Continuous | Years |
| 1. Biological sex | Binary | Male/Female |
| 1. Duration of current LBP episode | Continuous | Months |
| 1. Number of previous LBP episodes | Continuous | Number |
| 1. Number of other painful areas | Continuous | Number |
| 1. Work status | Binary | Off work or reduced hours/ At work |
| 1. Injury compensation for LBP | Binary | Injury compensation/ no compensation |
| 1. Highest level of education | Categorical (5 levels) | Year 10/ TAFE certificate/ Year 12 (HSC)/ Diploma/ Bachelor degree or higher |
| 1. Depression | Continuous | Depression subscale of the DASS-21^1^ |
| 1. Concern of LBP severity | Continuous | Reassurance Questionnaire^2^ |
| 1. Sleep difficulty | Continuous | Insomnia severity index^3^ |
| 1. Pain knowledge | Continuous | Neurophysiology of Pain Questionnaire^4^ |

^1^Lovibond PF, Lovibond SH. The structure of negative emotional states: Comparison of the Depression Anxiety Stress Scales (DASS) with the Beck Depression and Anxiety Inventories. *Behav Res Ther*. 1995;33(3):335-343. doi:10.1016/0005-7967(94)00075-U

^2^Speckens AEM, Spinhoven P, Van Hemert AM, Bolk JH. The reassurance questionnaire (RQ): Psychometric properties of a self-report questionnaire to assess reassurability. *Psychol Med*. 2000;30(4):841-847. doi:10.1017/S0033291799002378

^3^Bastien CH, Vallières A, Morin CM. Validation of the insomnia severity index as an outcome measure for insomnia research. *Sleep Med*. 2001;2(4):297-307. doi:10.1016/S1389-9457(00)00065-4

^4^Moseley L. Unravelling the barriers to reconceptualisation of the problem in chronic pain: The actual and perceived ability of patients and health professionals to understand the neurophysiology. *J Pain*. 2003;4(4):184-189. doi:10.1016/S1526-5900(03)00488-7
